# Myocardial Involvement Detected Using Cardiac Magnetic Resonance Imaging in Patients with Systemic Sclerosis: A Prospective Observational Study

**DOI:** 10.3390/jcm10225364

**Published:** 2021-11-18

**Authors:** Milan Hromadka, Jan Baxa, Jitka Seidlerova, Roman Miklik, Dan Rajdl, Vendula Sudova, David Suchy, Richard Rokyta

**Affiliations:** 1 Department of Cardiology, University Hospital and Faculty of Medicine in Pilsen and Faculty Hospital, Charles University, Alej Svobody 80, 304 60 Pilsen, Czech Republic; hromadka@fnplzen.cz (M.H.); rokyta@fnplzen.cz (R.R.); 2 Department of Imaging Methods, University Hospital and Faculty of Medicine in Pilsen, Charles University, Alej Svobody 80, 304 60 Pilsen, Czech Republic; baxaj@fnplzen.cz; 3 Internal Department II, University Hospital and Faculty of Medicine in Pilsen, Charles University, Edvarda Benese 1128/13, 305 99 Pilsen, Czech Republic; seidlerovaji@fnplzen.cz; 4 Department of Clinical Biochemistry and Hematology, University Hospital and Faculty of Medicine in Pilsen, Alej Svobody 80, 304 60 Pilsen, Czech Republic; rajdl@fnplzen.cz (D.R.); sudovave@fnplzen.cz (V.S.); 5 Department of Clinical Pharmacology, Rheumatology, University Hospital and Faculty of Medicine in Pilsen, Charles University, Edvarda Benese 1128/13, 305 99 Pilsen, Czech Republic; suchyd@fnplzen.cz

**Keywords:** systemic sclerosis, cardiac magnetic resonance, T1 mapping, galectin-3, GDF-15

## Abstract

Introduction and objectives: Cardiac involvement in systemic sclerosis (SSc) patients affects mortality. Cardiac magnetic resonance (CMR) is capable of detecting structural changes, including diffuse myocardial fibrosis that may develop over time. Our aim was to evaluate myocardial structure and function changes using CMR in patients with SSc without known cardiac disease during a 5-year follow-up and find possible correlations with selected biomarkers. Methods: A total of 25 patients underwent baseline and follow-up CMR examinations according to a pre-specified protocol. Standard biochemistry, five biomarkers (hsTnI, NT-proBNP, galectin-3, sST2, and GDF-15), and disease-specific functional parameters enabling the classification of disease severity were also measured. Results: After five years, no patient suffered from manifest heart disease. Mean extracellular volume (ECV) and T1 mapping values did not change significantly (*p* ≥ 0.073). However, individual increases in native T1 time and ECV correlated with increased galectin-3 serum levels (r = 0.56; *p* = 0.0050, and r = 0.71; *p* = 0.0001, respectively). The progression of skin involvement assessed using the Rodnan skin score and a decrease in the diffusing capacity of the lungs were associated with increased GDF-15 values (r = 0.63; *p* = 0.0009, and r = −0.51; *p* = 0.011, respectively). Conclusions: During the 5-year follow-up, there was no new onset of heart disease observed in patients with SSc. However, in some patients, CMR detected progression of sub-clinical myocardial fibrosis that significantly correlated with elevated galectin-3 levels. GDF-15 values were found to be associated with disease severity progression.

## 1. Introduction

Systemic sclerosis (SSc) is an immune-mediated rheumatic disease characterized by excessive extracellular matrix deposition, widespread fibrosis of the skin and visceral organs, microvascular injury, and immune system activation [1]. SSc can affect any cardiac structure, causing myocardial abnormalities such as myocardial fibrosis, myocardial microvascular ischemia, pericarditis, pericardial effusion, and less commonly, valvular disease. These may be clinically manifested by left ventricular systolic or diastolic dysfunction and bradyarrhythmias or tachyarrhythmias. It has been proven that cardiovascular involvement significantly contributes to the mortality of patients with SSc [2]. However, primary cardiac involvement is difficult to assess accurately for a variety of reasons. These reasons include the long asymptomatic course of the disease, various nonspecific symptoms associated with cardiac manifestations, the accuracy of the diagnostic method used, and the choice of defining criteria to characterize the disease [3,4]. 

Myocardial involvement can be demonstrated as a combination of myocardial ischemia and patchy fibrotic deposits, equally distributed throughout the left and right ventricular myocardium, with or without inflammation [5]. Myocardial damage is supposed to be caused by abnormalities in the coronary microcirculation, including vasospasms, recurrent ischemia-reperfusion injury, and impaired coronary vasodilation reserve, which is not associated with atherosclerotic coronary artery disease [4,5]. 

Cardiac magnetic resonance imaging (CMR) appears to be a reliable technique for anatomical and functional assessment of the heart, helping clinicians detect and analyze different mechanisms of cardiac involvement, including inflammatory, microvascular, and fibrotic processes [6]. In the early stages of the disease, myocardial fibrosis is rather diffuse and does not reach the detection limits of the late gadolinium enhancement (LGE). However, the technique of T1 mapping can assess diffuse myocardial processes [7]. The significant benefits of this method are pixel-based parametric imaging and the potential to quantify T1 relaxation on the basis of assessing the severity of cardiac involvement [8,9]. Native and post-contrast T1 mapping combined with LGE creates a comprehensive diagnostic approach capable of precisely quantifying the degree of myocardial fibrosis. 

Several biomarkers have been studied concerning myocardial involvement. Values of high sensitivity cardiac troponins and N-terminal pro-brain natriuretic peptides (NT-proBNP) are known to be elevated in SSc patients [10,11] and have been shown to be associated with heart involvement and outcomes [12]. Other biomarkers that could express the severity of the disease and degree of myocardial fibrosis have also been investigated [13,14]. 

The published data suggest that the development of myocardial fibrosis is a slow and silent process that can evolve outside the detection limits of standard biochemistry or ECHO [11,15,16]. To the best of our knowledge, no work has been published so far to assess the development of changes detected by CMR over longer times. After five years of follow-up, our work aimed to investigate CMR changes in SSc patients and correlate the results with various selected biomarkers and clinical findings. Furthermore, an observational sub-analysis of the effect of renin-angiotensin-aldosterone system (RAAS) inhibition on the dynamics of CMR parameters, biomarker values, and clinical status was performed. 

## 2. Methods

### 2.1. Study Population

This prospective study included 25 out of 33 patients with progressive systemic sclerosis who underwent a baseline study examination in 2015 [10]. In short, the patients were eligible for the study if none of the exclusion criteria was present: (1) history of heart disease and diabetes mellitus; (2) ECHO signs of pulmonary hypertension; (3) other heart rhythms than sinus rhythm; (4) contraindication for CMR including gadolinium allergy; (5) glomerular filtration < 30 mL/min; (6) pregnancy or breastfeeding. At the end of the 5-year follow-up, eight patients could not be re-evaluated (one pregnancy, two deaths due to a malignancy, one stroke, one non-participation due to immobility, two withdrawals of study consent; Figure S1 in Supplementary Material).

The study was approved by the Ethics Committee at the University Hospital in Pilsen. All participants signed informed consent for the study. Each subject underwent all study procedures in one day (blood sampling first, followed by CMR imaging).

### 2.2. CMR Protocol

Follow-up CMRs were performed using the same MRI scanner used for the baseline examinations. The 3.0 T device (MAGNETOM Skyra, Siemens Healthcare, Erlangen, Germany) was equipped with a 32-element surface coil for the thorax or a body coil. All sequences were performed during mild breath-holding with ECG synchronization (retrospective ECG gating technique). All measurements were done by an experienced radiologist who performed image analyses at baseline; however, the radiologist was blinded to any previous or current results. The protocol consisted of the following steps: (1) routine TrueFISP (True Fast Imaging with Steady-state Precession) sequence for morphological orientation and left ventricular function assessment in standard long-axis orientations (four-chamber and two-chamber); (2) pre-contrast (native) T1 maps; (3) dynamic (first-pass) perfusion; (4) TrueFISP sequence covering the whole left ventricle in short axis for functional analysis; (5) T1-weighted phase-sensitive inversion recovery (PSIR) sequence for detection of late gadolinium enhancement (LGE) and (6) post-contrast T1-maps. Native and post-contrast T1 maps were performed in three short-axis levels (basal, mid-ventricular, and apical) of the left ventricle. The sequence based on modified look-locker inversion recovery (MOLLI) with single shot TrueFISP was part of the commercially available package MyoMaps (Siemens Healthcare, Erlangen, Germany). T1 maps sequences were performed in the same levels using a single shot inversion recovery TrueFISP (fast imaging with steady-state free precession) with following parameters: TR 280.56 ms, TE 1.12 ms, echo spacing 2.7 ms, flip angle 35°, SL 8 mm, FOV 360 mm, matrix size 256 × 66%, voxel size 1.4 mm × 1.4 mm × 8 mm, iPAT 2. The native T1 maps were performed using a MOLLI type 5(3)3 sequence. Post-contrast T1 maps used MOLLI type 4(1)3(1) sequence and were performed with a minimal 15 min delay after the contrast agent (Gadovist; Schering, Berlin, Germany) was administered intravenously at 0.05 mmol/kg body weight [17]. The T2 sequence for edema detection was eliminated due to the low occurrence of edema at baseline.

### 2.3. CMR Analysis

Two independent radiologists, blinded to baseline images, performed all measurements in random order. Interobserver and intraobserver agreement of T1 value measurements were excellent using an intraclass correlation coefficient (0.94 for interobserver and 0.96 for intraobserver). T1 values analysis was performed using a region of interest (ROI) that was manually drawn in the intramyocardial part of the interventricular septum and the final T1 value was calculated as a mean of values from all three layers (basal, mid-ventricular, and apical). ROIs were carefully performed and the borders of the myocardium were not included. Regions of LGE were avoided to prevent influencing the final T1 value. The value of the extracellular volume (ECV%) fraction using native and post-contrast myocardial T1 values and hematocrit was calculated according to the following formula: [ECV (%) = (1 − hematocrit) × (1/post-contrast T1 of myocardium − 1/native T1 of myocardium)/1/post-contrast T1 of blood − 1/native T1 of blood) [10].

### 2.4. ECHO

All patients underwent a follow-up echocardiographic examination according to the baseline ECHO protocol [10], with the elimination of the speckle-tracking strain evaluation of the left ventricle.

### 2.5. Biomarkers

N-terminal pro-brain natriuretic peptide (NT-proBNP) was determined using original analytical kits from Roche (Prague division, Prague, Czech Republic) on a Cobas 8000 analyzer. High-sensitivity cardiac troponin I (hsTnI) was measured using the Architect i2000 platform with the STAT High Sensitive Troponin-I assay (Abbott Diagnostics, Abbot Park, IL, USA). Growth/Differentiation Factor-15 (GDF-15; RayBiotech, Norcross, GA, USA) and galectin-3 (MyBiosource, San Diego, CA, USA) concentrations were determined using ELISA kits on a Nexgen ELISA four reader (Adaltis, Rome, Italy). Suppression of tumorigenicity2 (sST2) was measured using a Critical Diagnostics Presage^®^ ST2 Assay kit (Critical Diagnostics, San Diego, CA, USA). Routine biochemical parameters including renal functions were also measured in all subjects and did not change over time.

### 2.6. Assessment of SSc Disease Severity

The severity of skin fibrosis was quantified using the modified Rodnan skin score (mRSS). The skin thickness at each of the 17 anatomical sites was classified from 0 to 3; the maximum score was 51. The carbon monoxide diffusing capacity of the lungs (DLCO) was measured using the single-breath method and a body plethysmograph Platinum elite™ (Medgraphic, Saint Paul, MN, USA).

### 2.7. Statistical Analysis

For statistical analysis, SAS software version 9.4 (SAS Institute Inc., Cary, NC, USA) was used. Changes in levels of biomarkers from baseline to the end of the follow-up were evaluated using the paired Wilcoxon test. Differences in biomarkers between groups were tested using the Mann–Whitney U test. The strength of relationships between examined biomarkers was measured using the Pearson correlation. Statistical significance was set at the level of α = 0.05.

## 3. Results

### 3.1. Characteristics of the Study Population

Our study population consisted of 25 SSc patients aged 55.5 years at baseline (range 26–71); 88% were women. The median duration of the disease was 10.0 years (range 2–40). The majority of patients (84%) suffered from the diffuse form. The median follow-up was 5.2 years (range 4.9–5.3). The proportion of patients using corticosteroids was 64% both at baseline and after 5 years of follow-up, and the use of disease-modifying anti-rheumatic drugs was 52% at baseline and 56% after 5 years of follow-up, which remained similar over time. 

As expected, the parameters reflecting pulmonary (DLCO) or skin progression (mRSS) of the disease worsened over the five years of follow-up (Table 1). However, none of the patients developed manifest or subclinical heart failure, new-onset arrhythmia, or met the criteria for pulmonary hypertension. Conventional echocardiographic parameters did not change. 

### 3.2. Cardiac Magnetic Resonance

Regarding CMR, neither mean values of native T1 relaxation time (*p* = 0.95) nor extracellular volume (*p* = 0.073) changed significantly over time. In addition to the parameters listed in Table 1, no abnormal changes were seen on the dynamic perfusion test. Concerning LGE changes over time, only one patient developed new lesions (10 patients with pathological LGE at baseline vs. 11 patients at follow-up).

### 3.3. Biomarkers and Their Correlation with CMR and Disease Activity 

**Figure 1:**
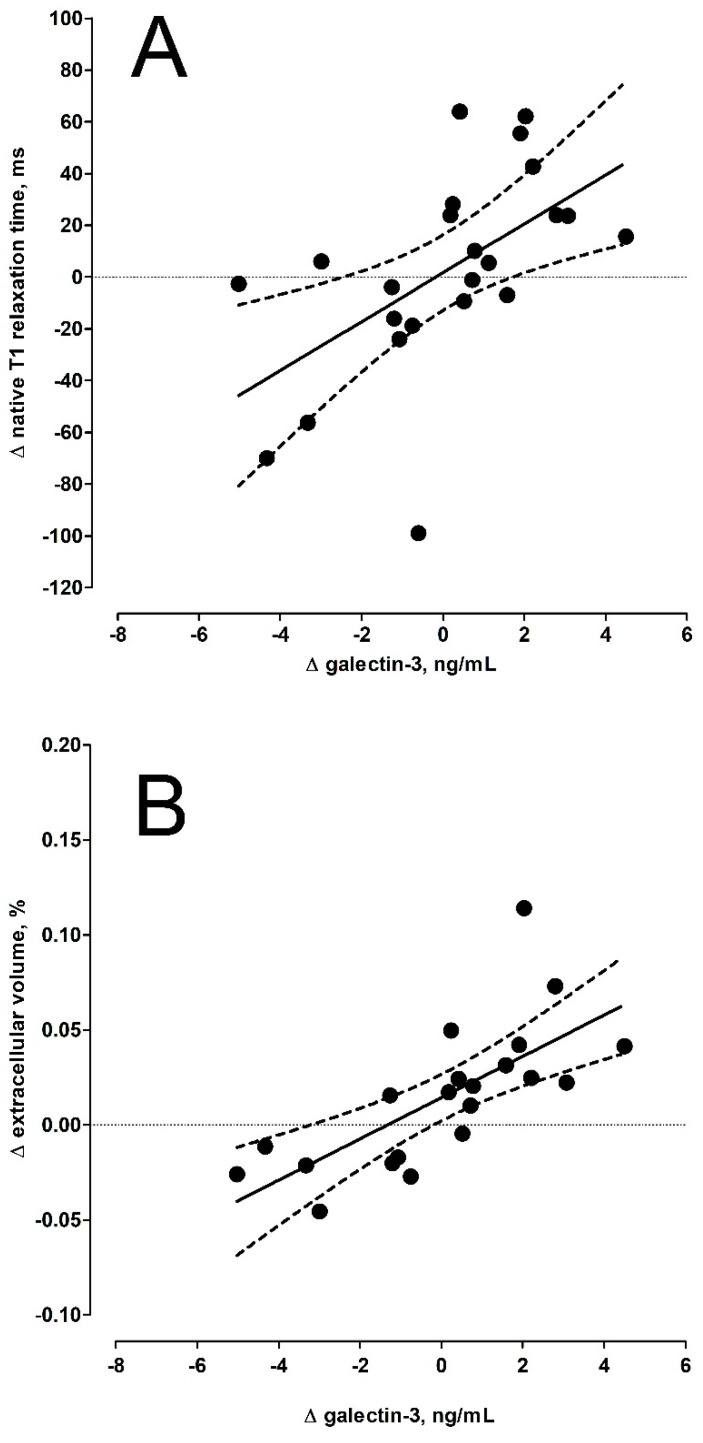
Relation between changes in galectin-3 levels and changes in native T1 relaxation time (panel (**A**)) and changes in extracellular volume (panel (**B**)).

Of the tested biomarkers, we observed a significant increase in sST2 (Δ +287.2 ± 507.2 ng/mL; *p* = 0.0078) and GDF15 (Δ +372.2 ± 631.9 ng/mL; *p* = 0.0046). Mean levels of hsTnI, NT-proBNP, and galectin-3 remained similar (Table 1). In a further step, we analyzed correlations between the change of the monitored biomarkers, CMR parameters, and disease severity examinations over time (per-patient analysis; Table 2 and Figure 1). Increased galectin-3 was associated with higher native T1 times (r = 0.56; *p* = 0.0050) and extracellular volumes (r = 0.71; *p* = 0.0001), while GDF-15 positively correlated with higher mRSS (r = 0.63; *p* = 0.0009) and negatively with lower values of DLco (r = −0.51; *p* = 0.011). Changes in hsTnI, NT-proBNP, or sST2 were not significantly related to changes in any of the monitored CMR parameters or clinical examinations. 

### 3.4. The Effect of RAAS

**Figure 2:**
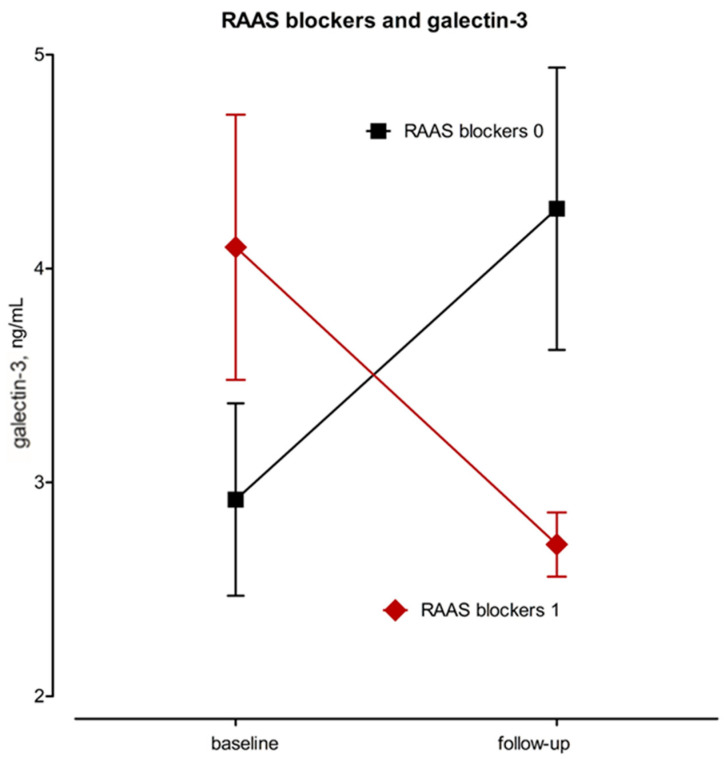
Galectin-3 values according to use of RAAS blockers. Values are means (standard errors of means). RAAS blockers = blockers of renin-angiotensin-aldosterone system.

Lastly, we investigated the effect of RAAS blockers on the change of biochemical and CMR parameters over time. In patients using an angiotensin-converting-enzyme inhibitor (ACEI) or sartan, the serum levels of galectin-3 decreased (Δ −1.31 ± 2.07 ng/mL), while in non-users, the values increased (Δ +1.35 ± 1.90 ng/mL; Table S1 in Supplementary Material and Figure 2). 

Furthermore, there was a smaller increase in sST2 in the RAAS blocker users compared to non-users (Δ +82.16 ± 484.61 ng/mL and Δ +492.37 ± 459.75 ng/mL; *p* = 0.0151), and a greater decrease in TnI values in the RAAS blocker users compared to non-users (Δ −3.71 ± 11.01 ng/L and Δ −2.20 ± 30.73 ng/L; *p* = 0.0227). No significant effect of RAAS blockade on CMR or other biochemical parameters was observed.

## 4. Discussion

The major findings of the study are as follows: (1) mean values of native T1 time and ECV detected by CMR in patients with systemic sclerosis did not change significantly during the 5-year follow up, but there are patients with CMR signs of progression or regression of the sub-clinical myocardial deterioration. These changes were significantly correlated with the dynamics of galectin-3. (2) The GDF-15 values were significantly correlated with the change of clinical scores of pulmonary or skin progression of the disease, thus potentially serving as a biomarker of the disease activity. (3) RAAS inhibitors did not show an effect on CMR parameters in patients without clinical signs of myocardial dysfunction but, when compared to RAAS non-users, were associated with either a decrease or a smaller increase of galectin-3, sST2, and hsTnI.

Concerning the CMR results in our group of patients, the baseline values of native T1 and ECV were already increased compared to healthy controls, thus confirming the presence of sub-clinical myocardial involvement [10]. Other studies have also confirmed the ability of CMR to quantify the early stages of fibrosis [8,9]. However, to the best of our knowledge, the presented study is unique in having performed serial CMR follow-up, evaluated changes of T1 mapping and ECV values in time, and detected remarkable changes of markers of fibrosis in both directions. In a large meta-analysis, cardiac involvement and the presence of pulmonary hypertension were found to be the strongest negative prognostic factors in SSc patients (hazard ratios of 4.35 and 5.27, respectively) [18]. Despite the fact that echocardiography is recommended as a screening tool [19], our results showed that a routine/standard ECHO (not including advanced modalities) could not detect the early fibrotic changes. ECHO can detect the presence of pulmonary hypertension, but that is a late sign of disease progression. Whether advanced speckle-tracking analyses, 3D volume calculations, or tissue doppler imaging may ameliorate the diagnostic power of echocardiography needs to be further determined. 

Concerning the dynamics of selected biomarkers, galectin-3 values well reflected the changes in native T1 relaxation time and ECV. This biomarker is a multifunctional protein implicated in various biological processes, including fibrosis, angiogenesis, cell differentiation and apoptosis, and immune activation, all of which are associated with the development of systemic sclerosis [20]. Galectin-3 was significantly correlated with the extent, variety, symptoms, and signs of SSc [21], particularly the extent of skin fibrosis [22,23]. Its potential role as a biomarker of sub-clinic myocardial fibrosis (assessed using CMR) was first demonstrated in our baseline study [14]. In the presented follow-up study, changes in galectin-3 values were well correlated with CMR measures of myocardial fibrosis, but not with changes in the overall clinical and functional status of the disease over the 5-year follow-up. More data are needed to confirm our findings, but it seems that galectin-3 serial testing might be a useful approach in screening for myocardial fibrosis in asymptomatic SSc patients. In animal models, the extent of myocardial fibrosis following myocardial infarction is well correlated with both myocardial and serum galectin-3 levels, and expression of myocardial galectin-3 was significantly increased [24]. 

We observed worsening of skin thickness and a decrease in pulmonary diffusing capacity over time in our patients. Contrary to previous studies [11,21,22], only GDF-15 correlated with these changes in our study. GDF-15 expression is induced during fibrosis development, and serum levels correlated strongly with clinical symptoms of lung fibrosis, which was the case in a previous study [25] as well as in our baseline study. NT-proBNP serves as both a universal cardiomarker and a good marker of pulmonary hypertension progression [26]. None of our patients developed pulmonary hypertension. TnI values were increased in the fibrous processes in the heart [11], but TnI levels may be affected by various cardiovascular risk factors, renal pathology, and inflammation [27]. For the reasons given, neither NT-proBNP nor hsTnI showed significant correlations with functional (DLco) or clinical (mRSS) parameters.

Another marker of fibrosis, the sST2, serves as a decoy receptor for interleukin-33 and negatively regulates anti-fibrotic pathways in the myocardium. It can induce fibrosis of pulmonary, hepatic, skin, renal, and pancreatic tissue [13]. Our study did not demonstrate its direct association with changes in skin and pulmonary function; however, the mean values significantly increased over time and were affected by RAAS blockade.

Recent papers demonstrated that RAAS pathways interact with the transformation of growth factor-beta to enhance or retard fibrotic processes in multiple organs. Inhibition of the classic RAAS pathway is a promising approach [28]. In the present study, we performed an observational analysis comparing RAAS-inhibitor users to non-users. An ACE inhibitor or sartan was indicated in 12 (48%) of our patients due to concomitant hypertension during follow-up. The minimal duration of medication use was 24 months. In a univariate analysis, galectin-3 best reflected the treatment with RAAS inhibitors (Figure 2). As demonstrated by Li et al., the expression of galectin-3 played an important role in the process of myocardial fibrosis in ischemic hearts, and the galectin-3 inhibitor, as well as the ACE inhibitor perindopril, significantly reduced this expression and thus myocardial fibrosis [24]. Since anti-proliferative medication was similarly distributed between RAAS users and non-users, the data suggest that galectin-3 might be a suitable candidate for monitoring the efficacy of RAAS inhibition in fibrotic processes, particularly in the myocardium. Aldosterone, another RAAS inhibitor, was not prescribed to any of the patients, but its effect on reducing galectin-3 levels and its effect on myocardial fibrosis has been documented [29]. The positive effect of RAAS inhibition on reducing troponin and sST2 values in patients with heart failure or coronary syndrome [30,31]—the processes involving inflammation, fibrosis, or scar formation—may explain the favourable changes in the levels of these biomarkers in SSc patients taking RAAS blockers in our study.

The study has specific limitations: (1) It is a single-center study with only a small number of patients and a relatively short follow-up. Due to the rare incidence of SSc and the slow disease progression, long-term follow-up of patients is very difficult for logistic reasons. (2) We did not evaluate the prognostic impact of our results; therefore, postulations of clinical recommendations regarding the frequency of CMRs or collection of biomarkers cannot be determined. (3) We did not perform any global peak systolic strain (GLPS) measurements during the follow-up ECHO examination due to logistic reasons and time constraints. (4) The selection of biomarkers was based on their availability in our institution and limited by financial constraints. (5) For disease severity assessment, we chose the most relevant and the most frequently disease-affected organs to follow and detect progression of the disease: skin (mRSS) and lungs (DLco). Although routine biochemistry values, capable of detecting renal or hepatic deterioration, remained consistent over time, no other findings from imaging or specialized SSc examinations were included in the study. Nevertheless, the majority of patients had a diffuse form of SSc, which was largely inclined to cause skin and pulmonary changes.

## 5. Conclusions

Cardiac magnetic resonance was able to detect subtle diffuse myocardial fibrosis in asymptomatic SSc patients during the follow-up. Deterioration of CMR parameters associated with fibrosis development correlated with an increase in serum galectin-3 levels. Skin impairment and a decrease in diffuse lung capacity were related to an increase in GDF-15. In an observation analysis, concomitant treatment with RAAS inhibitors best correlated with galectin-3 levels but also positively affected hsTnI and sST2 levels, suggesting that RAAS medication is involved in the modulation of the fibrotic process. Whether galectin-3 is a suitable candidate biomarker for detecting myocardial damage in SSc patients requires further investigations.

## Figures and Tables

**Table 1:** Changes of selected parameters over five years of follow-up.

	Baseline	Follow-Up	*p*
Systolic blood pressure, mm Hg	134.8 ± 17.4	135.6 ± 14.8	0.6800
Diastolic blood pressure, mm Hg	82.6 ± 9.5	82.0 ± 9.3	0.7200
BMI, kg/m^2^	26.1 ± 4.4	26.7 ± 4.5	**0.0090**
**Medication**			
Use of RAAS blockers	0 (0.0)	12 (48.0)	<0.001
Corticosteroids	16 (64.0)	16 (64.0)	1.0000
Anti-rheumatic drugs	13 (52.0)	14 (56.0)	0.9870
**Disease severity markers**			
mRSS, points	17.3 ± 4.3	17.9 ± 4.4	**0.0020**
DL_CO_, %	80.7 ± 15.1	77.7 ± 13.9	**0.0043**
**CMR**			
Native T1, ms	1253.4 ± 56.8	1251.5 ± 64.6	0.9500
ECV, %	0.28 ± 0.03	0.29 ± 0.04	0.0730
**ECHO**			
LVEF, %	63.1 ± 2.1	62.8 ± 2.5	0.5790
E/e’	9.26 ± 3.1	10.24 ± 4.8	0.3098
**Biomarkers**			
hsTnI, ng/L	3.0 (2.0–5.8)	2.8 (2.2–8.7)	0.4800
NT-proBNP, ng/L	141.88 ± 102.87	194.12 ± 218.46	0.6567
Galectin-3, ng/mL	3.45 ± 1.88	3.51 ± 1.80	0.7000
sST2, ng/mL	1358.6 ± 410.2	1645.8 ± 725.0	**0.0078**
GDF 15, pg/mL	1042 (791–1525)	1299 (1061–1843)	**0.0046**

BMI, body mass index; AAS blockers = blockers of renin-angiotentensin-aldosterone system; mRSS = modified Rodnan skin score; DLco = diffusing capacity of the lung for carbon monoxide; CMR = cardiac magnetic resonance; ECV = extracellular volume; ECHO = echocardiography; LVEF = left ventricular ejection fraction; E/e’ = early ventricular filling velocity/septal mitral anulus velocity; hsTnI = high sensitivity troponin I; NT-proBNP = N-terminal pro-brain natriuretic peptide; sST2 = soluble suppression of tumorigenicity 2; GDF-15 = growth differential factor 15. Values are mean ± standard deviation, number (percentage) or median (interquartile range). *p* for difference was calculated using the Wilcoxon test; statistically significant values (*p* < 0.05) are in bold.

**Table 2:** Correlations between changes in biochemical, CMR, and activity disease parameters.

Changes In	Native T1	ECV	DL_CO_	mRSS
hsTnI	0.14	0.05	−0.11	0.15
*p*	0.52	0.83	0.60	0.48
Galectin-3	**0.56**	**0.71**	−0.12	0.06
*p*	**0.0050**	**0.0001**	0.57	0.77
sST2	0.05	0.07	−0.09	0.14
*p*	0.83	0.75	0.68	0.51
GDF 15	0.23	0.25	**−0.51**	**0.63**
*p*	0.29	0.24	**0.011**	**0.0009**
NT-proBNP	−0.0739	0.0569	−0.1005	0.3819
*p*	0.73	0.79	0.63	0.059

ECV = extracellular volume; DLco = diffusing capacity of the lung for carbon monoxide; mRSS = modified Rodnan skin score; hsTnI = high sensitivity troponin I; sST2 = soluble suppression of tumorigenicity 2; GDF 15 = growth differential factor 15; NT-proBNP = N-terminal pro-brain natriuretic peptide. Values are Pearson correlation coefficients with *p* values of significance (*p* < 0.05 are in bold).

## Data Availability

The data of this study can be provided to researchers from the corresponding author upon request.

## Supplementary Materials

The following are available online at https://www.mdpi.com/article/10.3390/jcm10225364/s1, Figure S1: Study flow chart, Table S1: Changes of selected CMR and biochemical parameters between renin-angiotensin-aldosterone blocker users and non-users over the 5-year follow-up.
