# Supplementary material for: Myocardial Involvement Detected Using Cardiac Magnetic Resonance Imaging in Patients with Systemic Sclerosis: A Prospective Observational Study
Source: J Clin Med. 2021 Nov 18;10(22):5364. doi: 10.3390/jcm10225364 (PMC8620356; doi:10.3390/jcm10225364)
Supplement: Supplementary file 1 [file jcm-10-05364-s001.zip › jcm-1444759-supplementary.pdf]

Supplementary Materials:

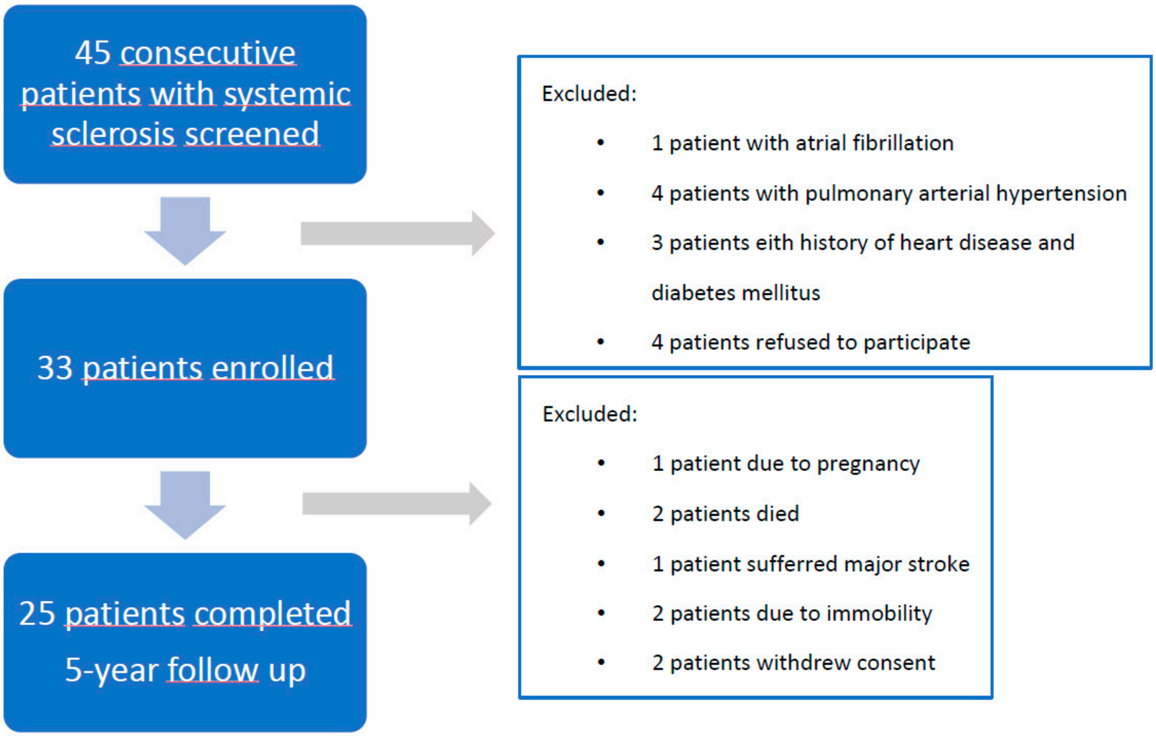

Figure S1. Study flow chart.

Table S1. Changes of selected CMR and biochemical parameters between renin-angiotensin-aldosterone blocker users and non-users over the 5-year follow-up.

**Changes in**	**RAAS blockers 0**<br>**n = 13**	**RAAS blockers 1**<br>**n = 12**	**P**
native T1, ms	4.28 ± 36.5	-8.47 ± 46.8	0.30
ECV, %	0.023 ± 0.025	0.005 ± 0.043	0.073
Galectin-3, ng/mL	**1.35 ± 1.90**	**-1.31 ± 2.07**	**0.0068**
GDF 15, ng/mL	522.8 ± 718.5	221.0 ± 518.8	0.30
sST2, ng/mL	**492.37 ± 459.75**	**82.16 ± 484.61**	**0.015**
hsTnI, ng/L	**-2.20 ± 30.73**	**-3.71 ± 11.01**	**0.023**
NT-proBNP, ng/L	130.30 ± 268.95	32.67 ± 135.25	0.16

RAAS blockers = blockers of renin-angiotensin-aldosterone system; ECV = extracellular volume; hsTnI = high sensitivity troponin I; sST2 = soluble suppression of tumourigenicity2; GDF 15 = growth differential factor 15; NT-proBNP = N-terminal pro-brain natriuretic peptide. Values are means of change (Δ)±standard deviation. P for difference between the two groups were calculated using Wilcoxon Two Sample test. P values < 0.05 are in bold.
